# A Lecture on Loxatrhus or Club-Foot

**Published:** 1841-04

**Authors:** 


Art. X.—*A Lecture on Loxarthus or Club-foot.* By T. D. Mütter, m.d., Lecturer on Surgery, Philadelphia.—*Philadelphia,* 1839. 8vo, pp. 104.

This lecture is very creditable to the author. It contains nothing new or original, but is a most accurate and methodic digest of the opinions of the various authors who have written on the subject of club-foot, tested by Dr. Mütter's own experience in about 30 cases. It would be a very useful book for students, wishing to make themselves acquainted with the theory and practical treatment of club-foot.

